# The genome sequence of the red-legged partridge, *Alectoris rufa* Linnaeus 1758

**DOI:** 10.12688/f1000research.162894.1

**Published:** 2025-09-02

**Authors:** Abderrahmane Eleiwa, Pol Mestre, Ana Usie, Ester Vilaprinyo, Alberto Marin-Sanguino, Albert Sorribas, Cristobal Richart, Judit Salces-Ortiz, Rosa Fernández, Mar Biarnés, Noélia Antilles, Jesus Nadal, Rui Alves

**Affiliations:** 1 Departament de Ciències Mèdiques Bàsiques, Universitat de Lleida, Lleida, Catalonia, Spain; 2 IRBLleida, Institut de Recerca Biomedica, Barcelona, Lleida, Spain; 3 Centre de Sanitat Avícola de Catalunya i Aragó (CESAC), Reus, Spain; 4 (MathSy2Bio) group 2021 SGR 01353, Mathematical Systems & Synthetic Biology in Biomedicine and Biotechnology, Lleida, Spain; 5 Centro de Biotecnologia do Alentejo (CEBAL), Beja, Portugal; 6 MED – Mediterranean Institute for Agriculture, Environment and Development and CHANGE – Global Change and Sustainability Institute, Beja, Portugal; 7 Universitat Rovira i Virgili, Tarragona, Catalonia, Spain; 8 Metazoa Phylogenomics and Genome Evolution Lab, Institute of Evolutionary Biology (CSIC-UPF), Barcelona, Spain; 9 Departament de Ciència Animal, Universitat de Lleida, Lleida, Catalonia, Spain

**Keywords:** Catalan Initiative for the Earth Biogenome Project, Birds, Phasianidae, Genome assembly, Reference Genome, Aves

## Abstract

The red-legged partridge ( *Alectoris rufa*) is a socio-economically important game bird in southern Europe. Despite previous efforts, achieving a high-quality, chromosome-level genome assembly has remained challenging. Here, we present a *de novo* phased, gapless reference genome of *A. rufa*, combining Nanopore long-read sequencing and Hi-C data from both sexes. The assembly resolves 40 nuclear chromosomes (38 autosomes and the 2 sex chromosomes, Z and W) and the mitochondrial genome, achieving chromosome-scale resolution and 99.1% completeness based on the Aves BUSCO dataset. This high-quality genome provides a critical resource for studying genetic diversity, sex-linked traits, and evolutionary adaptations.

## Introduction

The red-legged partridge ( *Alectoris rufa*) is a species of significant ecological and economic importance, particularly in southwestern Europe, ^1^ where it is a key game bird for hunting and rural economies. Despite its prominence, wild populations are declining due to habitat degradation and hunting pressure, leading to increased reliance on farm-reared partridges to meet hunting demands.

Limited insights into the bird’s evolution were obtained from the analysis of previous efforts that created assemblies with high to moderate genome fragmentation. ^1– 4^ That fragmentation limits our ability to fully explore the genomic basis of traits relevant to both wild and farmed populations, such as behavior, physiology, and adaptation.

Here, we present a *de novo* high-quality, chromosome-level genome assembly of *A. rufa*, incorporating both macro- and micro chromosomes, sex chromosomes, and the mitochondrial chromosome. We created this assembly through a hybrid approach combining long-read sequencing technologies (Oxford Nanopore) with Hi-C Illumina data, significantly improving contiguity, accuracy, and completeness over previous efforts. The inclusion of sex chromosomes provides a critical resource for understanding sex-linked traits and genetic diversity, which are essential for both conservation and breeding programs.

This chromosome-level assembly provides a foundation for identifying genetic markers associated with desirable traits, facilitating the development of genomic tools to ensure the genetic purity of wild partridges and enhancing the sustainability and productivity of farmed partridges.

## Methods

Table 1 provides the details on all software tools used for the assembly of the *A. rufa* genome.

**Table 1.** Software tools used.

Tool	Version	Parameters	Source
Poreshop	0.2.4	Default	https://github.com/rrwick/Porechop
Fastp	0.23.4	Default	8
NextDenovo	2.5.21	Default	21
NextPolish	1.4.0	Default	6
purge_haplotigs	1.1.3	-wind_min 10000	7
Gfastats	1.3.6	Default	15
BUSCO	5.7.1	-l aves_odb10	16
HapHiC	1.0.6	--correct_nrounds 2 max_inflation 10 --bin_size 200	9
Juicebox	1.6	Default	22
Ragtag	v2.1.0	Default	11
BlobToolKit	2.6.2	Default	17

### Sample collection

We randomly selected a wild female from Ciudad Real (central Iberian Peninsula) and a farm-raised male from Lleida (northeastern Iberian Peninsula). They were both anesthetized by inhaling isoflurane, before blood samples (0.25 ml) were drawn from the brachial vein of the wing using a sterile syringe with a 20G needle. After the procedure, the birds were allowed to recover and closely monitored for any signs of distress. The protocol was approved by the Ethics Committee on Animal Experimentation of the University of Lleida in 2016 (Ref. CEEA 09-06/16).

### Sample extraction, library construction and sequencing

High molecular weight (HMW) DNA was extracted from that blood for library preparation with the genomic DNA sequencing kit of Oxford Nanopore technology (ONT) as described in ^2^ and then sequenced the libraries using a GridION platform.

Chromatin conformation capture sequencing (Hi-C) libraries were prepared using the Hi-C High-Coverage kit (Arima Genomics) in the Metazoa Phylogenomics Lab (Institute of Evolutionary Biology [CSIC-UPF]). Sample concentration was assessed by Qubit DNA HS Assay kit (Thermo Fisher Scientific), and library preparation was carried out using the ACCEL-NGS 2S PLUS DNA LIBRARY KIT (Swift Bioscience) and using the 2S Set A single indexes (Swift Bioscience). We carried out library amplification with the KAPA HiFi DNA polymerase (Roche). The amplified libraries were sequenced on the NovaSeq 6000 (Illumina) with a read length of 2x151bp and a ~60Gb coverage, resulting in ~400M reads per library.

### Nuclear genome assembly

Nuclear genome assembly was performed using Nanopore raw data from one female and one male, as described in our previous work. ^2^ We implemented and used the Dorado base caller ^5^ to improve per-base quality of the original noisy ONT raw reads.

Assembly was carried out with NextDenovo pipeline. ^6^ The yielded contig-level assembly was polished using the NextPolish v1.4.0 ^6^ based on the ONT data. Haplotypic duplications were identified and removed using purge_haplotigs. ^7^ The purge_haplotigs contigs were used to filter out mitochondrial genome form the nuclear contig, as described below.

Fastp ^8^ was used to process the Hi-C removing low quality and duplication raw reads and HapHiC ^9^ was used on the Hi-C data to scaffolds the purged contigs. HapHiC is an allele-aware tool that enables scaffolding the haplotype-phased genome assembly into chromosome-scale pseudomolecules without the need for a preexistent reference genome. Chromosome assignment results were validated based on their synteny with respect to the *Gallus gallus* reference genome GCA_024206055.2, ^10^ using Ragtag v2.1.0. ^11^ Note that RagTag orders, orients, and joins sequences with gaps without altering the input query. We performed a final round of gap filling with Medaka ^12^ using the ONT raw reads. This was sufficient to generate the final gapless genome and capture the chromatin signal of both sex chromosomes of *A. rufa.* We independently assembled the male and female autosomes using sex-specific Hi-C data. We choose the autosome with the highest quality as the reference. However, due to the lower quality of the male Hi-C data, we used only female Hi-C data to assemble the sex chromosomes.

### Mitochondrial genome assembly and annotation

The mitogenome was extracted from the polished and purged nuclear contigs using a combined approach. First, the get_organelle_from_assembly.py script from GetOrganelle ^13^ was applied to the nuclear contigs, producing an uncircularized, gapped sequence. Next, Illumina short reads were used to circularize the mitogenome assembly. For annotation, MitoZ v3.6 ^14^ was employed. The genome assembly metrics were estimated using the Gfastats tool. ^15^ The BUSCO completeness score ^16^ was calculated within the BlobtoolKit2. ^17^

## Results and discussion

### Genome sequence report

**Figure 1.**
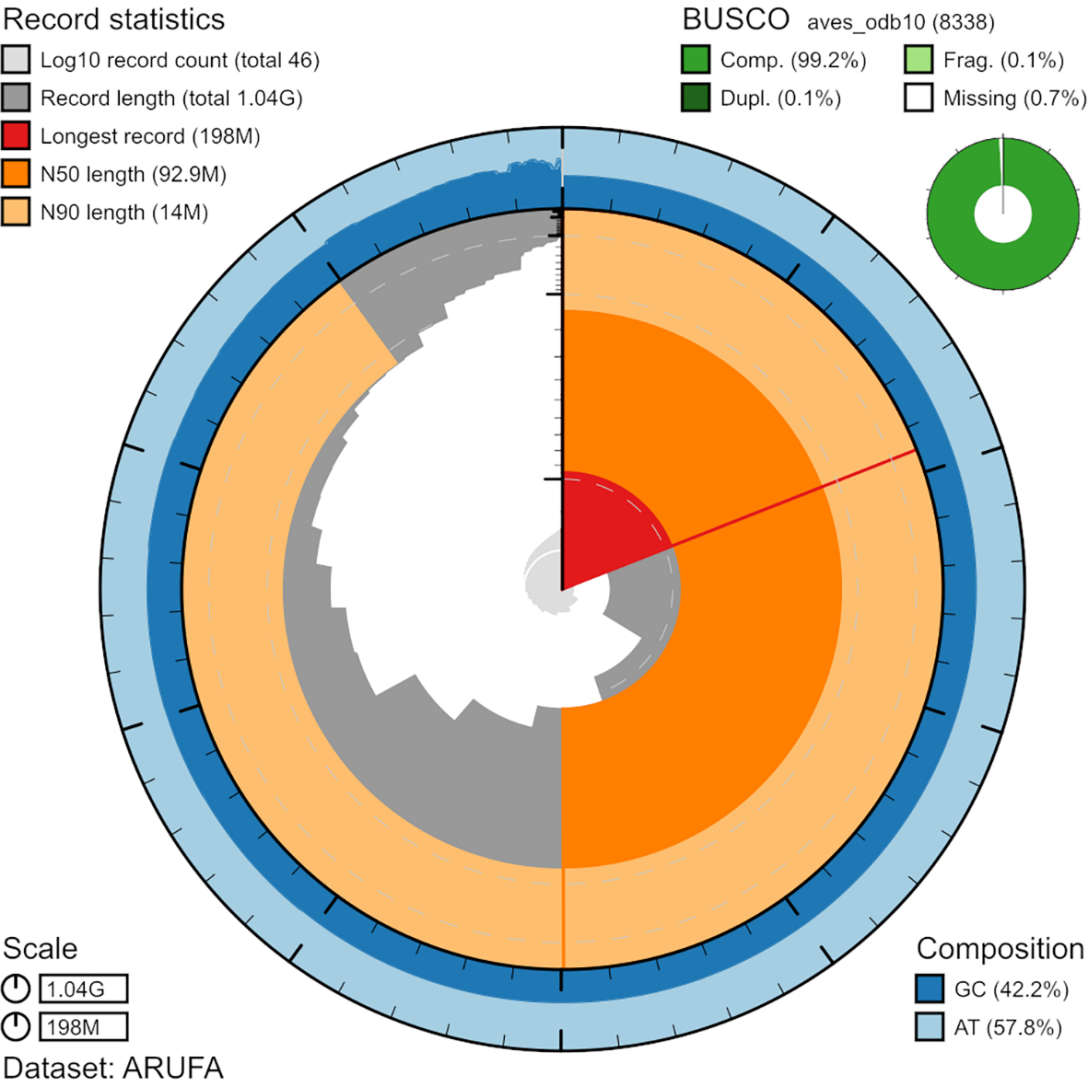
BlobToolKit Snailplot, representing the N50 metrics for *A. ruf*a assembly and BUSCO scores for the Aves set of orthologues. The distribution of chromosome lengths is shown in dark grey with the plot radius scaled to the longest chromosome present in the assembly shown in red.

**Figure 2.**
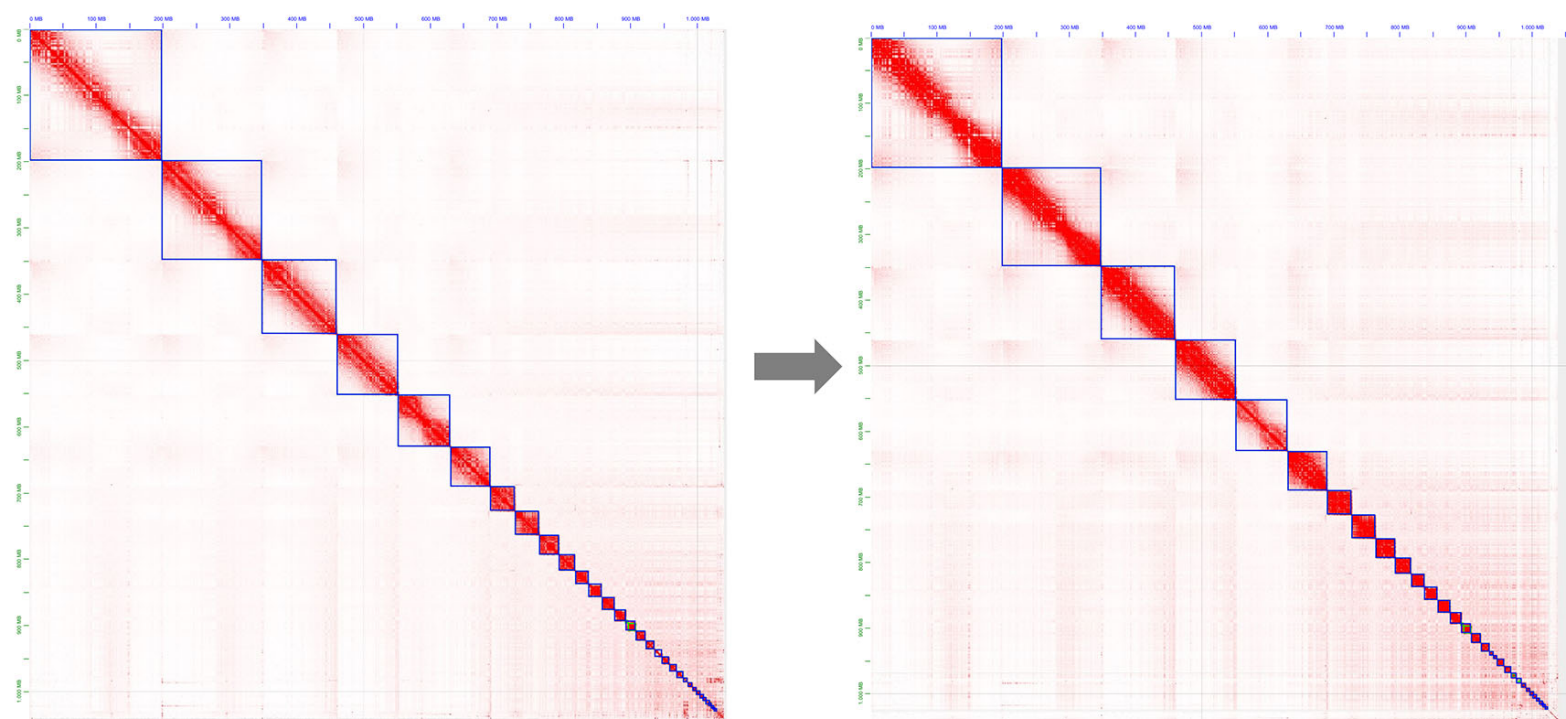
Juicebox visualization of the manually curated Hi-C contact map for the *A. rufa* assembly. Left-hand side: Original mapping. Right-hand side: Contact map after manual curation.

**Figure 3.**
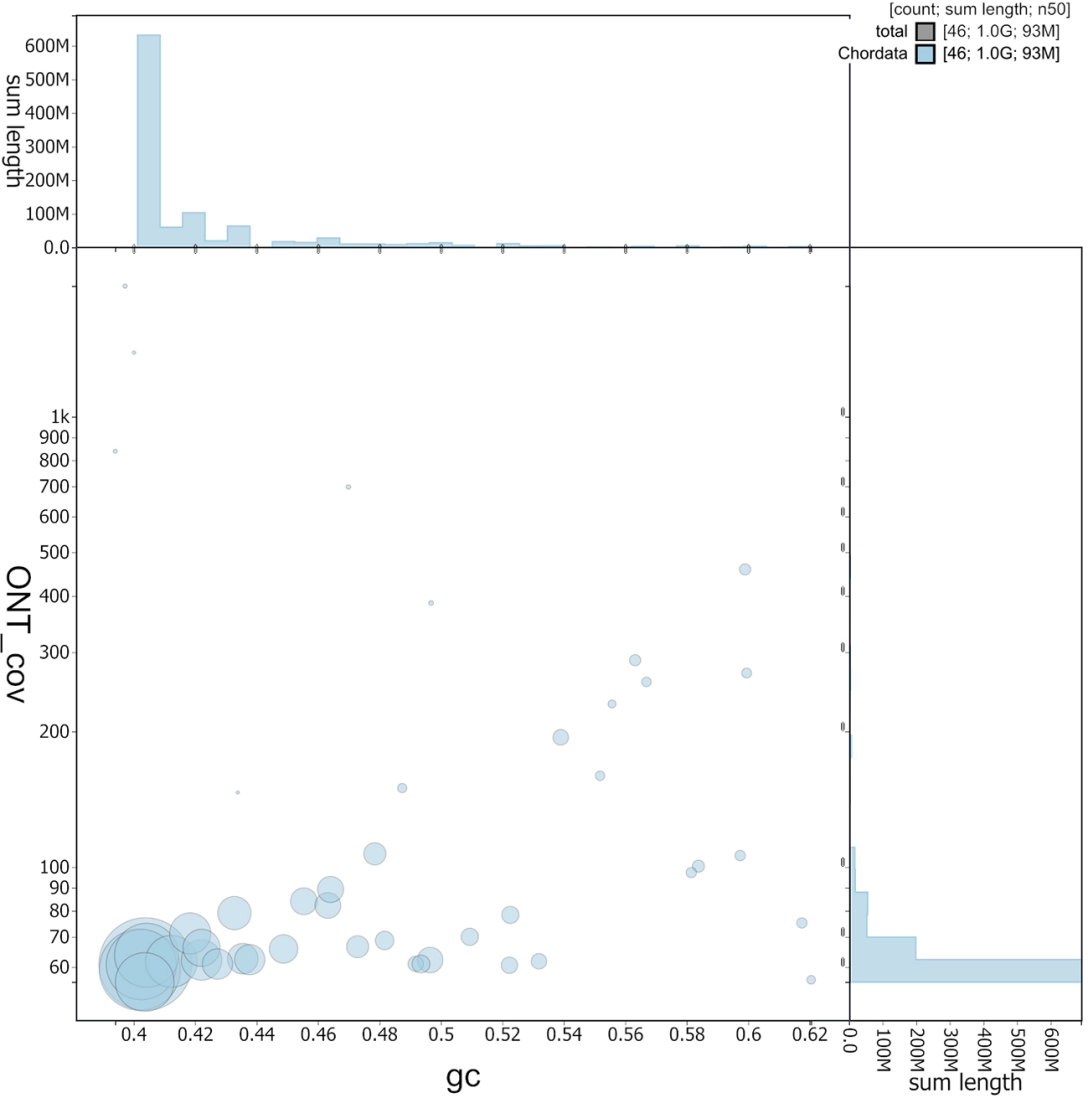
BlobToolKit GC-coverage plot. Circles are sized in proportion to scaffold length. Histograms show the distribution of scaffold length sum along each axis. Y-axis ontogene covariance (Chordata).

**Figure 4.**
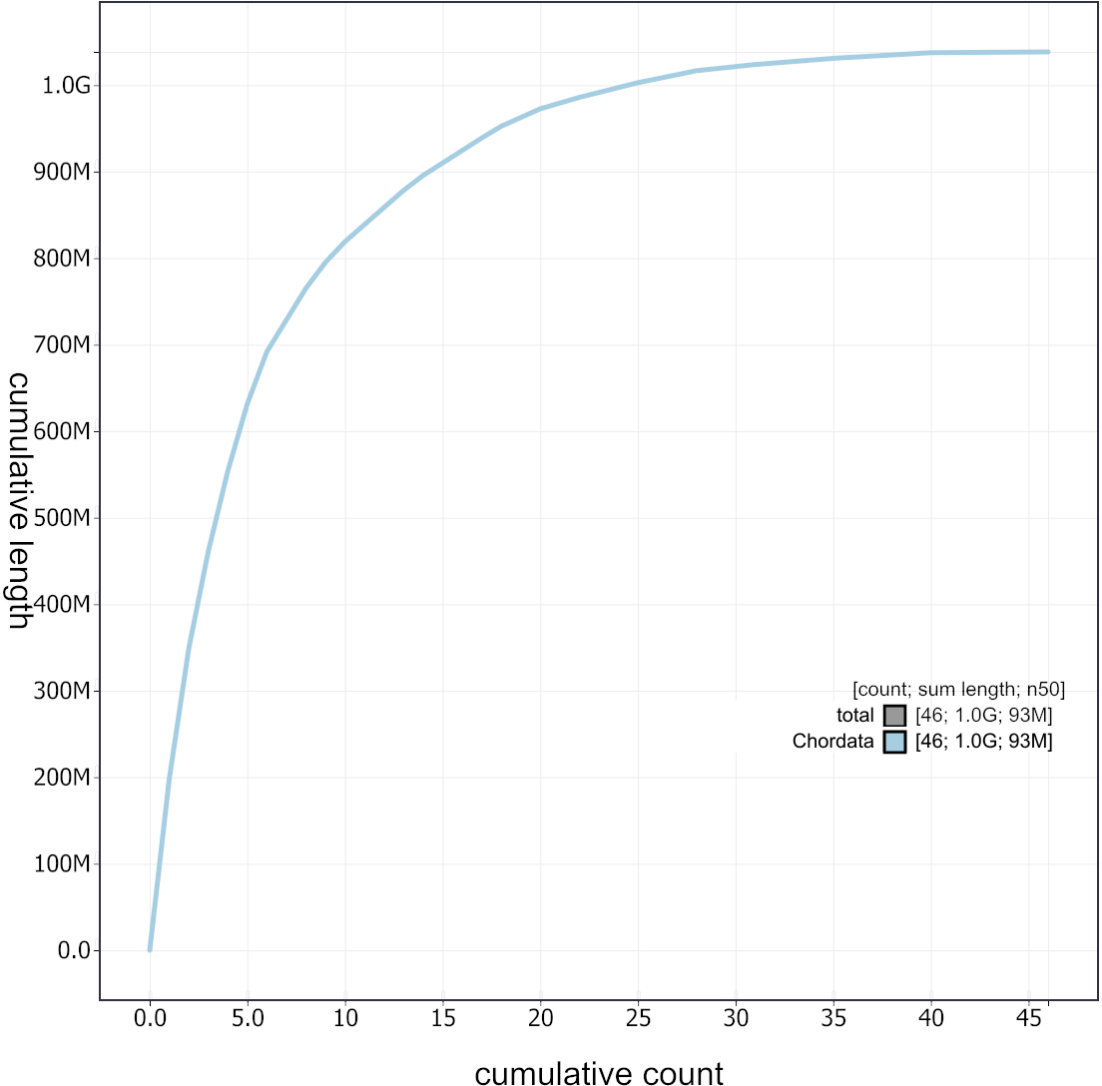
Cumulative sequence length plot of the *A. rufa* assembly. The grey line shows cumulative length for all sequences.

**Figure 5.**
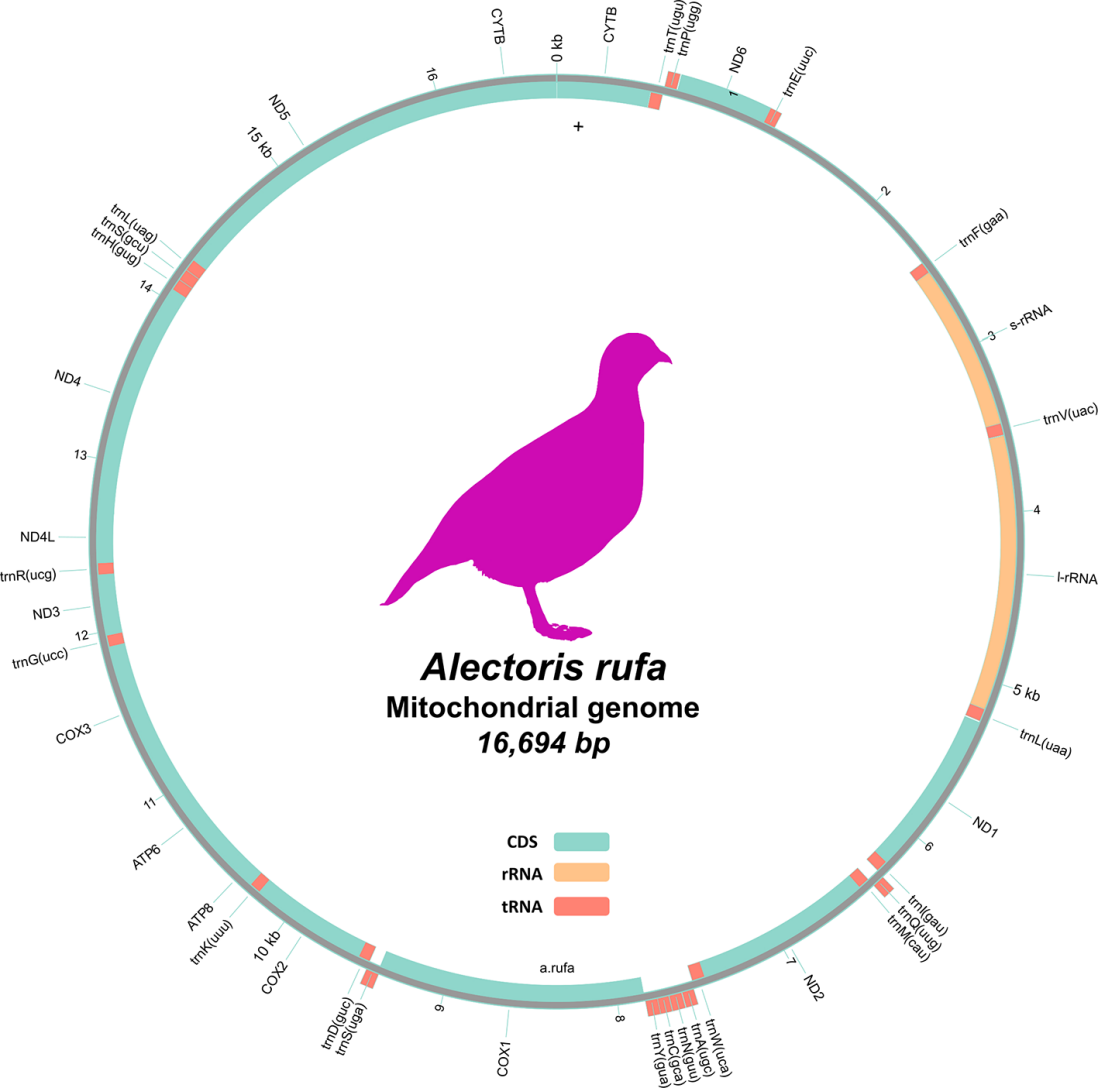
Mitochondrial genome gene map of *A. rufa.* The outermost track displays the annotated genes features. The middle track shows the coverage depth from ONT sequencing in 100 bp windows. The innermost track illustrates the GC content across the mitogenome, also in 100 bp windows.

We sequenced the genome of two *A. rufa* individuals, one male and one female. We summarize the statistics and deposition data in Table 2. We generated a total of 60-fold coverage data in Nanopore Ultra-long reads (N50 20 kb). We also generated 60-fold coverage data in Illumina NovaSeq6000 Hi-C sequencing. We scaffolded the primary assembly contigs from nanopore sequences with chromosome conformation HiC data. We also performed a final quality control step for the HiC scaffolds by validating them through synteny comparison to the *Gallus gallus* reference genome. The final assembly has a total length of 1.38Gb in 46 sequence scaffolds with scaffold/contig N50 of 92 Mb. 99.91% of the assembly sequence was assigned to 40 chromosomal-level scaffolds representing 38 autosomes plus the W and Z sex chromosomes ( Figures 1, 2, 3, and 4 and Table 2). The mitochondrial genome was 16.694 kbp long and contained 38 genes, including 22 tRNAs and 14 protein coding genes, with a GC percentage of 45.27 % ( Figure 5). The genome has a BUSCO completeness of 99.1% using the aves_odb10 reference dataset of orthologue single copy genes. The statistics for each chromosome, together with their ENA accession numbers are given in Table 3.

**Table 2.** Genome data for the *Alectoris rufa.*

*Project accession data*	
Assembly identifier	A_rufa_assembly2
Species	*Alectoris rufa*
Specimen	Arufa2401F
NCBI taxonomy ID	9079
BioProject	PRJNA1164475
Biosample ID	SAMEA114518447
Isolate information	Blood tissue (female & male)
*Raw data accessions*	
Nanopore raw data	ERR12165669, ERR12165668, ERR12165667, ERR12165666, ERR12165665, ERR14375934
Hi-C Illumina	ERR14648049, ERR14648048
*Genome assembly*	
Assembly accession	GCA_963854145
Span (Mb)	1,233
Number of contigs	46
Contig N50 length (Mb)	92
Number of scaffolds	46
Scaffold N50 length (Mb)	92
Longest scaffold (Mb)	198
BUSCO * genome score	C:99.1%[S:99.0%,D:0.1%], F:0.1%,M:0.8%,n:8338

* BUSCO scores based on the aves_odb10 BUSCO set using v5.7.1. C = complete [S = single copy, D = duplicated], F = fragmented, M = missing, n = number of orthologues in comparison.

**Table 3.** Chromosomal pseudomolecules in the genome assembly of *Alectoris rufa.*

*ENA accession*	*Chromosome*	*Size (bp)*	*GC%*	*ENA accession*	*Chromosome*	*Size (bp)*	*GC%*
OZ238766.1	Chr01	198016086	40.38	**OZ238786.1**	Chr021	6832952	48.17
OZ238770.1	Chr02	150934939	40.20	**OZ238787.1**	Chr022	4537674	49.18
OZ238768.1	Chr03	112339401	40.25	**OZ238788.1**	Chr023	5891266	50.94
OZ238769.1	Chr04	92881468	40.42	**OZ238789.1**	Chr024	6361550	49.35
OZ238770.1	Chr05	60168630	41.23	**OZ238790.1**	Chr025	993629	55.57
OZ238771.1	Chr06	36086843	42.21	**OZ238791.1**	Chr026	5251705	52.23
OZ238772.1	Chr07	37189634	41.84	**OZ238792.1**	Chr027	5836382	52.26
OZ238773.1	Chr08	29910164	42.21	**OZ238793.1**	Chr028	4642635	53.19
OZ238774.1	Chr09	23946095	43.28	**OZ238794.1**	Chr029	1487215	56.69
OZ238775.1	Chr010	20032715	43.55	**OZ238795.1**	Chr030	2250405	59.90
OZ238776.1	Chr011	19678379	42.73	**OZ238796.1**	Chr031	2219045	56.32
OZ238777.1	Chr012	19532764	43.78	**OZ238797.1**	Chr032	1812957	61.75
OZ238778.1	Chr013	17277913	44.88	**OZ238798.1**	Chr033	2650367	58.38
OZ238779.1	Chr014	15001852	45.54	**OZ238799.1**	Chr034	1468626	55.18
OZ238780.1	Chr015	13971621	46.32	**OZ238800.1**	Chr035	1892499	59.74
OZ238781.1	Chr016	4664116	53.90	**OZ238801.1**	Chr036	1635412	59.95
OZ238782.1	Chr017	10178771	47.85	**OZ238802.1**	Chr037	1157391	62.05
OZ238783.1	Chr018	13644036	49.65	**OZ238803.1**	Chr038	1811234	58.15
OZ238784.1	Chr019	9980371	47.29	**OZ238804.1**	Chr0W	1397749	48.74
OZ238785.1	Chr020	14276749	46.41	**OZ238805.1**	Chr0Z	77739472	40.36
				**OZ238806.1**	Chr0M	16694	45.27
					unplaced	914206	43.19

## Reporting guidelines

Figshare: ARRIVE Checklist for the Genome Note “The genome sequence of the red-legged partridge, Alectoris rufa Linnaeus 1758”, https://doi.org/10.6084/m9.figshare.29501561.v1 ^23^

This project contains the following data:

ARRIVE Author Checklist - E10 only.pdf

Data are available under the terms of the Creative Commons Attribution 4.0 International license (CC-BY 4.0).

## Data Availability

ENA Chromosome Sequences: All sequence data is deposited at the ENA, under accession numbers GCA_963854145.2, OZ238766.1, OZ238770.1, OZ238768.1, OZ238769.1, OZ238770.1, OZ238771.1, OZ238772.1, OZ238773.1, OZ238774.1, OZ238775.1, OZ238776.1, OZ238777.1, OZ238778.1, OZ238779.1, OZ238780.1, OZ238786.1, OZ238787.1, OZ238788.1, OZ238789.1, OZ238790.1, OZ238791.1, OZ238792.1, OZ238793.1, OZ238794.1, OZ238795.1, OZ238796.1, OZ238797.1, OZ238798.1, OZ238799.1, OZ238800.1, OZ238801.1, OZ238802.1, OZ238803.1, OZ238804.1, OZ238805.1, OZ238806.1, OZ238781.1, OZ238782.1, OZ238783.1, OZ238784.1, and OZ238785.1. NCBI raw data sequences: Nanopore raw read data are available via NCBI (Bioproject accession numbers PRJNA1050768, Biosample accession numbers: ERS16499794, ERS16499793, ERS16499792, ERS16499791, ERS16499790, SRX23440923). Links to the data: https://www.ncbi.nlm.nih.gov/bioproject/1050768 ^18^ https://www.ncbi.nlm.nih.gov/nuccore/2724812281 ^18^ https://www.ncbi.nlm.nih.gov/sra/SRP486622 ^19^ https://www.ebi.ac.uk/ena/browser/view/GCA_963854145.2 ^20^
