# Supplementary material for: The genome sequence of the red-legged partridge, Alectoris rufa Linnaeus 1758
Source: F1000Res. 2025 Sep 2;14:855. [Version 1] doi: 10.12688/f1000research.162894.1 (PMC12464528; doi:10.12688/f1000research.162894.1)
Supplement: Peer review history

# Reviewer report: Reviewer response for version 1

**DOI:** 10.5256/f1000research.179167.r412834

**Published:** 2025-09-25

**Authors:** Greta Bellinzona

**Affiliations:** 1 University of Pavia, Pavia, Italy

The manuscript by Eleiwa et al. reports a chromosome-level assembly of the red-legged partridge ( *Alectoris rufa*), a species of ecological and economic importance. The methods integrate long-read Nanopore sequencing with Hi-C Illumina data and produce a genome with good contiguity and completeness. The study makes a valuable contribution to the genomic resources available for Galliformes.

 While the importance is clear and the genome is of good quality (based on the statistics reported by the authors), the presentation of the Methods and Results would benefit from a bit of clarification:

 1. *Hi-C library preparation*: The workflow is described in detail but without sufficient clarity on whether both individuals were used for Hi-C or just one (later text implies only female Hi-C was used for sex chromosomes, is that correct?)

 2. Methods, Nuclear genome assembly. “haplotype-phased genome assembly” is claimed for HapHiC scaffolding, but it is unclear whether phasing was retained in the final assembly. Clarify whether the assembly is a haploid representation, phased, or partially phased

 3. The coverage values are inconsistently reported: “~60 Gb coverage” in Methods vs. “60-fold coverage” in Results. Please clarify the exact sequencing depth (fold coverage vs. Gb generated).

 4. The mitogenome section states “38 genes” but later specifies “22 tRNAs and 14 protein coding genes”: this adds up to 36. Were 2 rRNAs omitted in the description?

 5. Figure 3.  What the “ontogene covariance (Chordata)” axis represents? Add details in the caption.

 6. The protocol approval is included, but it would be good, if possible, to mention adherence to relevant guidelines for wildlife sampling (e.g., minimizing harm, use of anesthesia)

 Minor edits:

 - filter out mitochondrial genome "form" the nuclear contig” -- “from”

 - “to scaffolds the purged contigs” -- “scaffold"

Are the datasets clearly presented in a usable and accessible format, and the assembly and annotation available in an appropriate subject-specific repository?

Yes

Are sufficient details of the sequencing and extraction, software used, and materials provided to allow replication by others?

Yes

Are the rationale for sequencing the genome and the species significance clearly described?

Yes

Are the protocols appropriate and is the work technically sound?

Yes

Reviewer Expertise:

Genomics, parasitology

I confirm that I have read this submission and believe that I have an appropriate level of expertise to confirm that it is of an acceptable scientific standard.

# Reviewer report: Reviewer response for version 1

**DOI:** 10.5256/f1000research.179167.r412828

**Published:** 2025-09-19

**Authors:** Marc Palmada-Flores

**Affiliations:** 1 University of Girona, Girona, Spain

In this article the authors describe and make publicly available a chromosome-level genome assembly of the reg-legged partridge ( *Alectoris rufa*). The use of HiC data and ONT data allowed to reach a much better contiguity and completeness than previously fragmented references.

 Firstly, I want to congratulate the authors on the clear description of the reasons why they sequenced this species and the methodology used.

 I have several questions and comments regarding the paper:

 1- In the first paragraph of the introduction you mention a hunting pressure and habitat degradation without citing any paper that support this claim, although habitat degradation might be common knowledge I am reluctant to think hunting is currently a big pressure to this specific species of partridge, could you cite any paper or document that can support these claims?

 2- In the second paragraph of the introduction you give a great reason to generate a chromosome-level genome assembly. You could compare the resulting assembly to the previously available fragmented genomes to make clear that point in the present paper and even support the claims by comparing the results of a test analyses using one assembly or the other. 

 3- In the first paragraph, second line of the nuclear genome assembly section, basecaller is written jointly, not seperately as you do.

 4- In the second paragraph, first line of the nuclear genome assembly section, I would not describe NextDenovo as a pipeline, but rather as a tool and the citation is wrong, it should be changed to the one you have as 21.

 5- In the second paragraph, second line of the nuclear genome assembly section, it would be great to know which ONT mapping procedure you used to be able to replicate the polishing step.

 6- In the third paragraph of the nuclear genome assembly section, I do not understand why you use Ragtag if you already performed an scaffolding reference-free step, it would be great to see the differences between the scaffolded and the rescaffolded assemblies to value this extra step.

 7- In the third paragraph of the nuclear genome assembly section, I think it would be more clear if we could know how many gaps and contigs/scaffolds you had in every step, prior to scaffold, after first scaffolding with HapHiC, second scaffolding with RagTag and after the gap filling with Medaka. 

 8- In the third paragraph of the nuclear genome assembly section, using RagTag and gap filling are not as common as other steps in the genome assembly at chromosome-level of vertebrates. It would be valuable to have more insights into the outputs and value of these two steps.

 9- In the third paragraph of the nuclear genome assembly section, you write Ragtag v2.1.0. and then RagTag, for consistency I would stick with RagTag. Also in Table 1 I think you should change it to keep consistency.

 10- In the mitochondrial genome assembly and annotation section the version, database and annotation software used to check BUSCO completeness in BlobtoolKit2 should be mentioned.

 11- In the results' genome sequence report section I miss some explanation related to having 0 gaps in the resulting scaffolds (which are actually contigs) and would change to talk to contigs instead because you no longer have any kind of gaps.

 Overall, I think this work should be indexed and made a great and good effort to achieve the chromosome-level genome reference for this species.

Are the datasets clearly presented in a usable and accessible format, and the assembly and annotation available in an appropriate subject-specific repository?

Yes

Are sufficient details of the sequencing and extraction, software used, and materials provided to allow replication by others?

Partly

Are the rationale for sequencing the genome and the species significance clearly described?

Yes

Are the protocols appropriate and is the work technically sound?

Yes

Reviewer Expertise:

genomics, genome assembly, reference genomes, population genomics, conservation genomics, biodiversity genomics, bioinformatics

I confirm that I have read this submission and believe that I have an appropriate level of expertise to confirm that it is of an acceptable scientific standard.
